# John Charlton

**Published:** 1823-05

**Authors:** 


### OBITUARY.

On the 1st of April, aged 85, JOHN CHARLTON, Esq. upwards of twenty years surgeon-major to the 1st, or Grenadier Regiment of Foot Guards. Mr. Charlton had passed more than half a century in the service of his country. He commenced his career as surgeon to the 17th regiment of Foot, at the siege of the Havannah, in the year 1760; and had subsequently served in the West Indies, in the French American war, in the expedition against the Cherokee Indians, and lastly in the American war of independence. He was for many years attached to the suite of his late Royal Highness the Duke of Gloucester, brother to his Majesty George III., whom he accompanied on his travels to the continent; and, by his skill and unremitting attention, was chiefly instrumental in recovering his Royal Highness from a most severe and dangerous illness, which he experienced whilst in Italy. There were few places on the continent of Europe which Mr. C. had not visited, and his mind was stored with a fund of information and anecdote, which rendered his conversation and society a source of continued and varied amusement. Professionally, he was a sound and judicious practitioner; in the intercourse of social and domestic life, a most amiable man, of the strictest integrity, and possessed of the keenest feelings of honour. His memory will long live in the recollection of a numerous circle of friends and acquaintance, who fully appreciated his worth, and whose consolation must now be to look back to his long, honourable, and useful career.

---

